# Supplementary material for: Multiple Secondary Healthcare-Associated Infections Due to Carbapenem-Resistant Organisms in a Critically Ill COVID-19 Patient on Extensively Prolonged Venovenous Extracorporeal Membrane Oxygenation Support—A Case Report
Source: Microorganisms. 2021 Dec 23;10(1):19. doi: 10.3390/microorganisms10010019 (PMC8781848; doi:10.3390/microorganisms10010019)
Supplement: Supplementary file 1 [file microorganisms-10-00019-s001.zip › Supplementary materials.pdf]

Supplementary materials

Supplementary Method 1. We collected environmental samples with eSwab 480 CE (Copan, Brescia, Italy) and incubated them with 10 mL of Mueller–Hinton broth at 37 °C for 48 h for pre-enrichment. Then, we placed 100 μL of the broth on the CHROMagarTM mSuper CARBA (Nippon Becton Dickinson, Tokyo, Japan) and incubated the sample at 37 °C for 48 h.

Supplementary Method 2. Both *Klebsiella aerogenes* isolates KE-1 and KE-2 were confirmed to be negative for carbapenemase genes of $bla_{\text{IMP-1}}$, $bla_{\text{IMP-6}}$, bla_{VIM}, bla_{GES}, bla_{KPC}, bla_{NDM}, and $bla_{\text{OXA-48}}$ groups by using Cica geneusR Carbapenemase Genotype detection kit 2 (Kanto Chemical CO., Inc., Tokyo, Japan).

Supplementary Method 3. Whole genome sequencing (WGS) was performed on both *Klebsiella aerogenes* isolates KE-1 and KE-2 as follows: bacterial DNA was extracted with the QIAmp DNA Mini GIAcube Kit (Quiagen, Hilden, Germany) according to the manufacturer's instructions, and a DNA library was prepared from each sample with a Nextera XT DNA Sample Prep Kit (Illumina, San Diego, CA, USA) according to the manufacturer's instructions. For sequencing, a Miseq (Illumina, San Diego, CA, USA) was used to generate paired-end 300 bp reads. Both samples showed an average of 150-fold coverage (Supplementary Dataset). Phylogeny based on WGS-based core-genome

multilocus sequence typing (cgMLST) was performed with SeqSphere + version 6.0.2 (Ridom GmbH, Münster, Germany) by using an ad hoc scheme comprising 4466 target loci based on the KCTC2190 genome (NC_015663.1) (Jünemann S et al., Nat Biotechnol 2013; Seth-Smith HMB et al., Front Public Health 2019). The *K. aerogenes* isolates KE-1 and KE-2 were genetically separated by 71 cgMLST allele differences (Weber DJ et al., Curr Opin Infect Dis 2013).

Supplementary Dataset. Total numbers of contigs, total length, N50 length, average coverage, and accession numbers for *Klebsiella aerogenes* isolates KE-1 from blood culture and KE-2 from perianal abscess drainage culture analyzed with whole-genome sequencing. Both samples showed an average of 150-fold coverage. The sequence data have been deposited in the DDBJ/EMBL/GenBank Sequence Read Archive under accession numbers DRX279247 and DRX279248.
